# Oxalate of Cerium in Pregnancy

**Published:** 1879-05-20

**Authors:** Francis Edward Image


### *ON THE EMPLOYMENT OF OXALATE OF CERIUM IN PREGNANT SICKNESS.*

BY DR. FRANCIS EDWARD IMAGE, M.A.

Sir James Simpson introduced the oxalate of cerium, and prescribed it in ten-grain doses. The officinal dose is from one to two grains, which is as a rule so useless that the preparation has been stigmatized as the "oxalate of mud." As a general practitioner of seven years standing, very many cases of pregnant sickness have naturally come under my care, and up to the present time I have not met with a case in which the nausea has not been very considerably relieved, and in most cases completely checked by ten-grain doses of the oxalate of cerium. I have, at the time of writing this, a lady under my care, who, from the fourth week of her pregnancy till now, the eighth month, has suffered at intervals from this distressing symptom, but whose sickness has been invariably checked by from two to three days' administration of the oxalate in the dose I have mentioned. In severe cases I give it every four hours for the first day, beginning the first dose half an hour before the patient rises, and then, as improvement takes place, diminishing it to three times a day, but always giving the first dose of the day before the patient moves from the horizontal position—a point to which I attach much importance. The formula I employ is ;

| | |
|---|---|
| Cerii oxalatis.................................... | x grs. |
| Pulv. trag. co.................................... | x grs. |
| Tinct. aurantii.................................... | ʒss. |
| Aquam ad .................................... | ℥i. M f.m. |

In Dr. Frowert's case, he prescribed 1½-grain doses, which were not followed by the slightest remission of symptoms. I hold that this want of good result was from the insufficiency of the dose.

The oxalate of cerium I have also found most efficacious in restraining the nausea resulting from uterine irritation. I generally combine it with bromide of potash in these cases, but have found it succeed in combination where the previous employment of the latter drug by itself has been without appreciable effect.—*Practitioner.*

